# Retrograde signaling is required for Slm35-mediated negative regulation of mitophagy in yeast

**DOI:** 10.1242/bio.062106

**Published:** 2026-01-07

**Authors:** Hilario Ruelas-Ramírez, Ariann E. Mendoza-Martínez, P. Abril Medina-Flores, Soledad Funes

**Affiliations:** Departamento de Genética Molecular, Instituto de Fisiología Celular, Universidad Nacional Autónoma de México, Coyoacán, Mexico City 04510, Mexico

**Keywords:** Mitophagy, Mitochondrial retrograde signaling, Yeast, Mitochondria, Atg32

## Abstract

Mitophagy is essential for mitochondrial quality control, selectively removing damaged or superfluous mitochondria to maintain cellular health and metabolic homeostasis. While positive regulators of mitophagy are relatively well characterized, the mechanisms governing its downregulation remain less understood. In this study, we investigate the role of *Saccharomyces cerevisiae* Slm35 – a protein previously involved in oxidative stress response – in the regulation of mitophagy. We discovered that Slm35 is a soluble mitochondrial matrix protein and functions as a novel negative regulator of mitophagy and the mitochondrial retrograde (RTG) signaling pathway. Our results show that Slm35 modulates mitophagy through the RTG pathway, independently of Atg32 proteolytic processing by Yme1 or mitochondrial membrane potential dissipation. Notably, Slm35 is crucial for the dynamic regulation of the RTG pathway in mitophagy-inducing conditions. These findings highlight the importance of Slm35 in fine-tuning mitochondrial quality control in response to metabolic cues and suggest a critical role for dynamic RTG pathway regulation in mitophagy control.

## Teaser

**Summary:** This study describes how the mitochondrial protein Slm35 works as a negative regulator of mitophagy, acting through retrograde signaling to the nucleus, and highlights its role in fine-tuning mitochondrial quality control.

## INTRODUCTION

To maintain mitochondrial quality control, cells rely on processes such as fusion, fission, and mitophagy. Mitophagy is a form of selective autophagy that enables the degradation of mitochondria through their recognition by either the PINK1-Parkin pathway (Geisler et al., 2010; Narendra et al., 2008, 2010) or via specific autophagy receptors, including BNIP3L/NIX (Novak et al., 2010), BCL2L13 (Murakawa et al., 2015), BNIP3 (Hanna et al., 2012), FUNDC1 (Liu et al., 2012), AMBRA1 (Strappazzon et al., 2015), FKBP8 (Bhujabal et al., 2017), and Prohibitins (Wei et al., 2017). All of these receptors, as well as the PINK1-Parkin pathway, have been identified in metazoans, with the exception of Prohibitins, which are also conserved in yeast (García-Chávez et al., 2024). These receptors are mitochondria-associated proteins that target them to the core components of the autophagic machinery. In the yeast *Saccharomyces cerevisiae*, Atg32 is the key mitophagy receptor (Kanki et al., 2009a; Okamoto et al., 2009) and facilitates the anchoring and fusion of vesicles, leading to the elongation of a membranous structure known as the phagophore around the mitochondrial portion to break down.

Atg32 is a single-span outer mitochondrial-membrane protein that exposes soluble domains toward both the intermembrane space (IMS) and the cytosol, with the N-terminus facing the cytosol (Kanki et al., 2009a; Okamoto et al., 2009). Its structure contains two important domains: AIM and A11BR (Farré and Subramani, 2016). The first, named after Atg8-family Interacting Motifs, consists of a conserved amino acid sequence (W/F/Y)XX(L/I/V) carrying at least one proximal acidic residue. The first and fourth amino acids interact directly with Atg8, while the acidic residues provide a negative charge that also contributes to the interaction. The A11BR region (Atg11 Binding Region), contains the amino acid sequence I/VLS, which enables binding to Atg11. The Atg32-Atg11 interaction allows Atg11 to recruit numerous components of the autophagic machinery, facilitating phagophore assembly around those mitochondria targeted for degradation (Kanki et al., 2009a).

The presence of Atg32 alone is not sufficient to initiate mitophagy, since several post-translational modifications are also required. For example, its phosphorylation is triggered by nitrogen starvation or by prolonged respiration in the stationary phase. This post-translational modification is carried out by casein kinase 2 (CK2) (Kanki et al., 2013) on serines 114 and 119 of the A11BR domain and is mediated by the factors Pbs2 and Hog1. Phosphorylation is required for Atg32-Atg11 interaction to occur (Aoki et al., 2011). The Ppg1 phosphatase counteracts CK2-dependent phosphorylation of Atg32 and acts as a negative regulator of mitophagy (Furukawa et al., 2018). Another important post-translational modification for mitophagy induction is the proteolytic processing of the carboxyl terminus of Atg32 by Yme1, an intermembrane space protease from the iAAA family (ATPase family associated with various cellular activities). Removal of the carboxyl terminus induces a conformational change in the cytosolic end that enhances its interaction with Atg11. Therefore, the lack of Yme1 protease or inhibition of its activity results in a significant defect in mitophagy, since the unprocessed form of Atg32 shows lower affinity for Atg11 (Wang et al., 2013).

While mitophagy is often seen as a cellular ‘last resort’ to eliminate irreversibly damaged mitochondria, its role in *S. cerevisiae* is less straightforward. Yeast cells lacking mitophagy show no major growth defects, even when exposed to conditions that typically trigger this process (Kanki et al., 2009a). Surprisingly, it is not the mitochondrial oxidative stress, but rather the lack of glutathione, that seems to induce mitophagy in this unicellular model organism (Deffieu et al., 2009). This suggests that other factors may be involved in targeting mitochondria for degradation. One such factor could be the mitochondrial retrograde signaling pathway (RTG), which adapts cellular metabolism to decreased tricarboxylic acid cycle (TCA) activity by translocating the transcription factors Rtg1 and Rtg3 to the nucleus, albeit only the latter has the transcription activation domain. There, they regulate the expression of genes involved in replenishing precursors. While a decrease in mitochondrial membrane potential (MMP) is known to trigger the retrograde response (Miceli et al., 2012), the exact mechanism by which this signal induces mitophagy remains elusive. Furthermore, although the activation of the retrograde pathway and mitophagy correlate in time, the precise interplay between these processes is not fully understood.

The Slm35 protein has been identified as one of the mitochondrial factors negatively regulating mitophagy. Initially identified as the product of the *SLM35* (*YJR100C*) gene in a large-scale study that established the first mitochondrial proteome (Reinders et al., 2006), Slm35 has been implicated in various organellar processes, including biogenesis and maintenance of the mitochondrial DNA (mtDNA) (Hess et al., 2009), cellular stress responses, and longevity (Aguilar-Lopez et al., 2016). Notably, when *SLM35* is not expressed, mitophagy increases; this led to the proposal that Slm35 is a negative regulator of this degradative process. Additionally, impaired maintenance and integrity of the mitochondrial network could be linked to the absence of *SLM35* and other components of the stress response pathways, such as *TORC1* (Aguilar-Lopez et al., 2016).

In this study, we show that Slm35 is a soluble protein residing in the mitochondrial matrix that functions as a negative regulator of mitophagy. Furthermore, we reveal that Slm35 negatively modulates the RTG signaling pathway. Our data indicate that Slm35 regulates mitophagy through the RTG pathway, acting independently of the proteolytic processing of Atg32 by Yme1 and without altering global mitochondrial membrane potential. Notably, our findings highlight that Slm35 plays a crucial role in the dynamic regulation of the RTG pathway in response to nutrient stress, suggesting its involvement in fine-tuning mitochondrial quality control.

## RESULTS

### Slm35 is a soluble mitochondrial matrix protein

To understand the role of Slm35 in mitophagy, we first investigated its precise sub-mitochondrial location. Although it was clear that Slm35 resides in the organelle (Hess et al., 2009; Reinders et al., 2006), some reports suggested it could localize either in the inner mitochondrial membrane (IMM) (Vögtle et al., 2017) or in the matrix (Morgenstern et al., 2017).

**Fig. 1.**
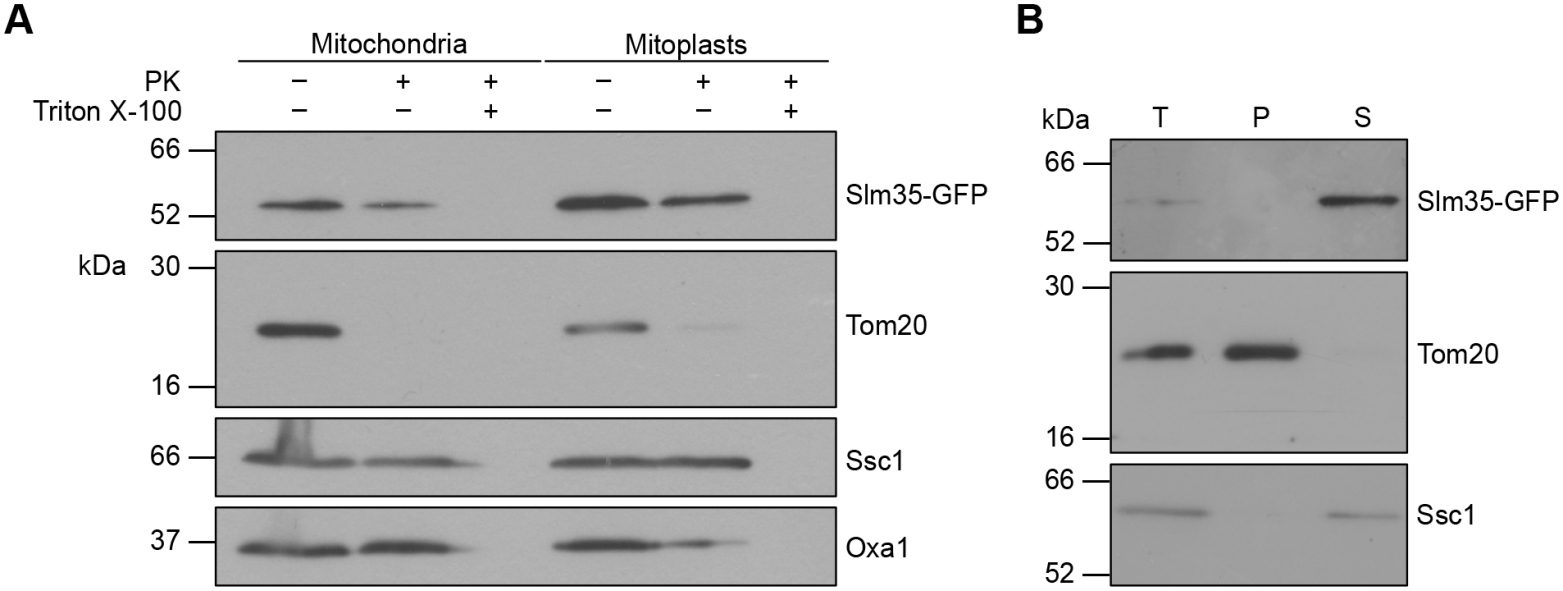
**Slm35 is a soluble mitochondrial matrix protein.** (A) Equal amounts of crude mitochondrial extracts (50 μg) from a strain expressing *SLM35-GFP* were treated with proteinase K (PK) and Triton X-100 in isotonic (mitochondria) or hypotonic (mitoplasts) conditions, as indicated. Samples were analyzed by western blotting using antibodies against GFP, Tom20 (OMM), Ssc1 (matrix), or Oxa1 (IMM facing the IMS). (B) Mitochondria from the *SLM35-GFP* expressing cells were treated with sodium carbonate (Na_2_CO_3_) to release soluble and membrane-associated proteins by ultracentrifugation. 10% of the mitochondrial material (50 µg) was analyzed as the total (T) sample, while the remaining 90% (450 µg) was fractionated into pellet (P; containing integral membrane proteins) and supernatant (S; containing soluble and membrane-associated proteins). The samples were then analyzed by western blotting using antibodies against GFP (Slm35), Tom20 (membrane protein) and Ssc1 (soluble protein). The blots shown are representative of *n*=3 independent biological replicates.

To identify the protein, we used cells expressing the *SLM35* gene from its endogenous locus to produce Slm35 with a green fluorescent protein (GFP)-tagged version at its carboxyl terminus. We isolated mitochondria from these transformants and studied the intramitochondrial localization using a proteinase K protection assay. As shown in Fig. 1A, treatment of mitochondria with this protease caused degradation of the outer mitochondrial membrane (OMM) protein Tom20 but not of the matrix protein Ssc1 nor of Slm35-GFP. Treatment under hypotonic conditions, which disrupts the OMM but maintains the IMM, prevented the degradation of both Ssc1 and Slm35-GFP. When all mitochondrial membranes were solubilized with Triton X-100, the protease could access all compartments and degraded all three proteins.

Next, we separated the membrane and soluble proteins of mitochondria using sodium carbonate treatment followed by ultracentrifugation. We found that Slm35-GFP was released into the supernatant, along with Ssc1 (Fig. 1B). Based on these results, we conclude that Slm35-GFP is a soluble protein of the mitochondrial matrix.

Additionally, protein structure predictions suggest that Slm35 has a β-barrel structure (Fig. S1A), which is typically found in OMM proteins and not in IMM proteins (Eaglesfield and Tokatlidis, 2021). When comparing the surface hydrophobicity of the AlphaFold-predicted (Abramson et al., 2024) structure of Slm35 with the experimentally determined structure of Tom40 (Wang et al., 2024), a known β-barrel protein in the OMM, Tom40 shows distinct hydrophobic patches (in brown) that are absent in Slm35 (Fig. S1B). Taken together, these results confirm the submitochondrial localization of Slm35 within the mitochondrial matrix, thereby providing a basis for further investigation into its role in mitophagy.

### Slm35 negatively modulates mitophagy induced by prolonged respiration in the stationary phase and by nitrogen starvation

To verify that *SLM35* negatively regulates mitophagy (Aguilar-Lopez et al., 2016), we manipulated Slm35 levels by overexpressing the *SLM35* gene from a 2μ vector, with an *ADH1* promoter, and measured mitophagy under standard laboratory induction conditions. We monitored mitophagy by tracking the degradation of Idh1-GFP, a protein located in the mitochondrial matrix. When mitophagy is induced and mitochondria are transported to the vacuole for degradation, Idh1-GFP is broken down by vacuolar proteases, generating free GFP.

**Fig. 2.**
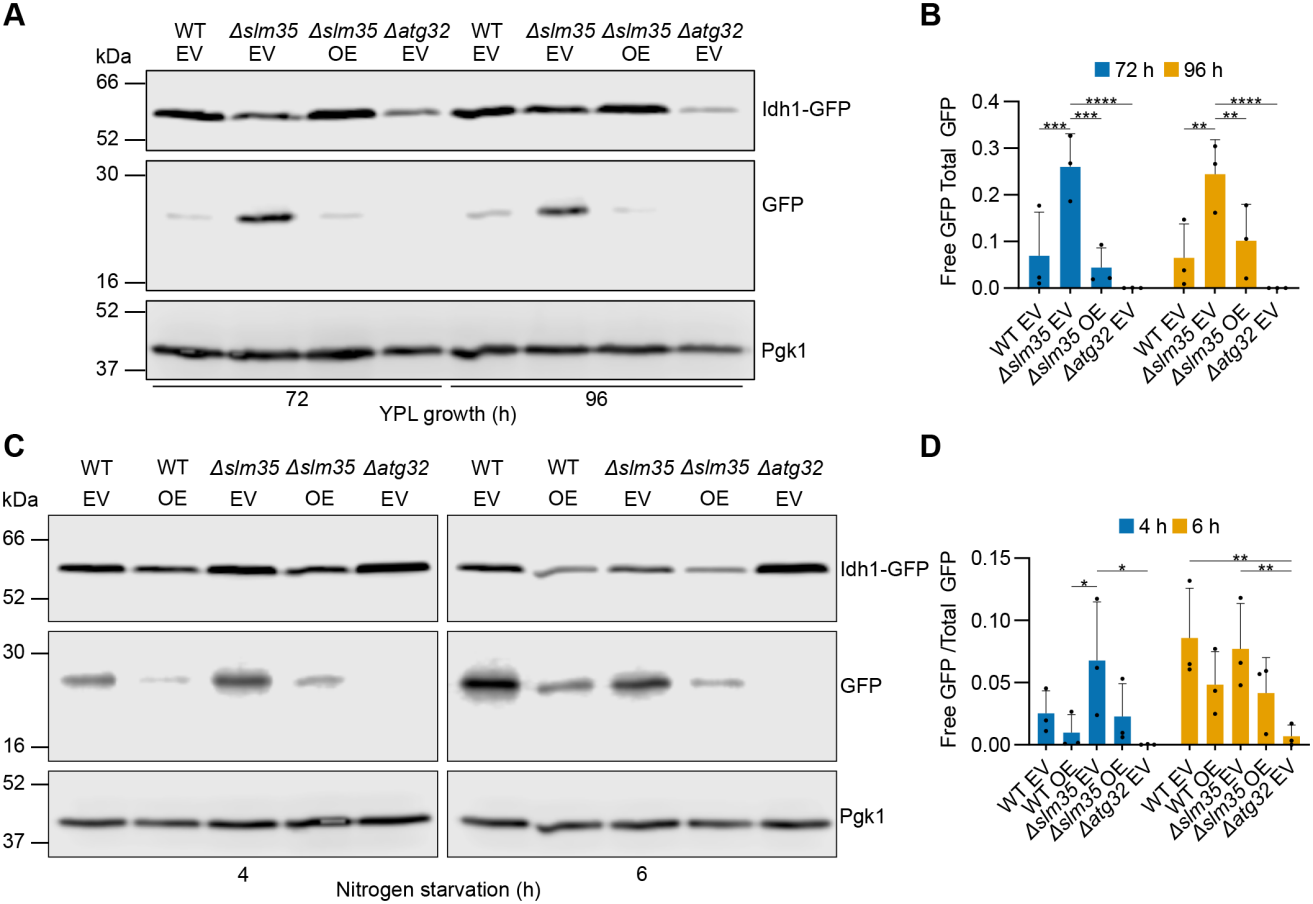
***SLM35* negatively regulates mitophagy.** (A) Cells expressing the *IDH1-GFP* mitophagy reporter were grown in YPL medium, and total protein extracts were collected at the indicated time points. Samples were analyzed by western blotting using antibodies against GFP and Pgk1. (B) Densitometric quantification of free GFP relative to total GFP signal from the blots shown in A. (C) Cells expressing the same reporter were subjected to nitrogen starvation, and protein extracts were collected at the indicated time points and analyzed as in A. (D) Densitometric quantification of free GFP relative to total GFP signal from the blots in C. Values represent mean±s.d. of *n*=3 independent biological replicates. Statistical significance was assessed using a two-way ANOVA followed by Tukey's multiple comparisons test. Significance levels: *P<0.05; **P<0.01; ***P<0.001; ****P<0.0001. Western blots in panels A and C were performed three times from independent replicates, and one representative blot is shown. WT: wild-type strain. EV: empty vector. OE: overexpression (*pVT100U-SLM35-7HIS*).

Mitophagy is induced by prolonged respiration in the stationary phase (lactate); the process starts around 48 h and is well established at 72 and 96 h, as indicated by the presence of free GFP in the wild-type strain. Deletion of *SLM35* consistently increased the amount of free GFP while overexpressing *SLM35-7HIS* in this mutant strain decreased free GFP levels (Fig. 2A). The densitometric quantification confirmed the increase in *Δslm35* cells and demonstrated a statistically significant decrease upon *SLM35-7HIS* overexpression (Fig. 2B).

Nitrogen starvation also triggered mitophagy starting at around 4 h; however, in contrast to what was observed in Fig. 2A, neither deletion of *SLM35* nor overexpression of *SLM35-7HIS* in both WT and *Δslm35* backgrounds, showed statistically significant changes of free GFP signals at 4 and 6 h (Fig. 2C,D).

These findings suggest that Slm35 protein levels are relevant during modulation of mitophagy induced by prolonged respiration in the stationary phase (lactate): depletion of *SLM35* levels increases mitophagy, while overexpression decreases the process. Furthermore, it supports the idea that *SLM35* negatively modulates mitophagy induced by either prolonged respiration in the stationary phase, but not by nitrogen starvation.

### Slm35 influences mitophagy through a pathway independent of Atg32 levels or proteolytic processing by Yme1

**Fig. 3.**
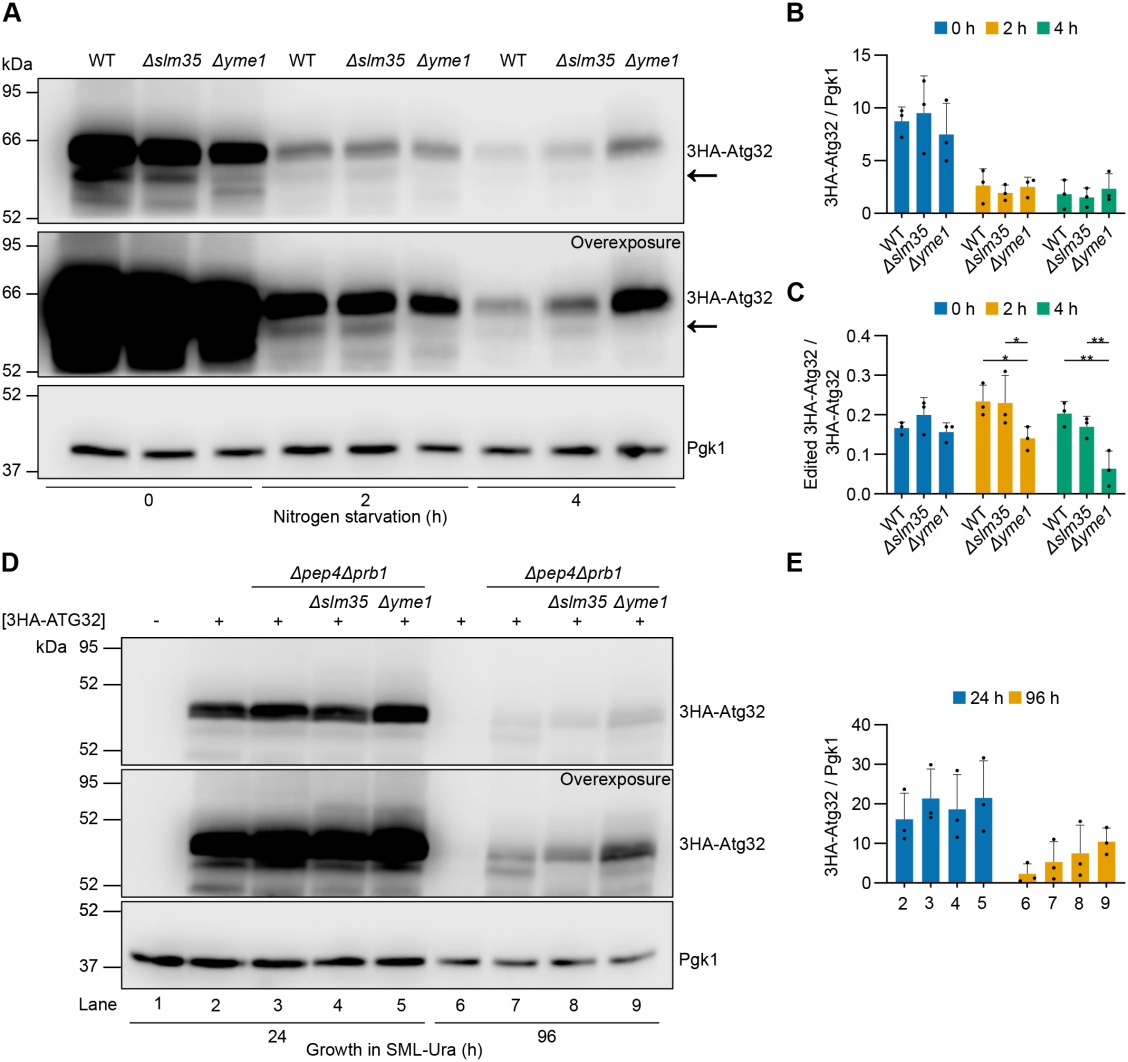
***SLM35* deletion did not result in the accumulation of edited 3HA-Atg32.** (A) Cells lacking *ATG32* and either *SLM35* or *YME1*, and expressing *3HA-ATG32* from a centromeric plasmid under its endogenous promoter, were subjected to nitrogen starvation. Protein extracts were collected at the indicated time points and analyzed by western blotting using antibodies against HA and Pgk1. The arrow indicates the processed form of 3HA-Atg32 that depends on Yme1. (B,C) Densitometric quantification of full-length (B) and edited (C) 3HA-Atg32 relative to Pgk1 and 3HA-Atg32, respectively. (D) Cells expressing *3HA-ATG32* as in (A), but lacking *PEP4* and *PRB1* and either *SLM35* or *YME1*, were grown in SML medium lacking Uracil. Protein extracts were collected at the indicated time points and analyzed by western blotting using antibodies against HA and Pgk1. (E) Densitometric quantification of 3HA-Atg32 relative to Pgk1. Values represent mean±s.d. of *n*=3 independent biological replicates. Statistical significance was assessed using a two-way ANOVA followed by Tukey's multiple comparisons test. Significance levels: *P<0.05; **P<0.01. Western blots in panels A and D were performed three times from independent replicates, and one representative blot is shown. WT: wild-type strain.

Both Atg32 protein levels (Kanki et al., 2009a) and proteolytic editing by Yme1 (Wang et al., 2013) induce mitophagy. Given that Slm35 negatively regulates mitophagy, we examined whether this effect involves alterations on Atg32 abundance or on its proteolytic processing by Yme1. Thus, we monitored the levels of 3HA-Atg32 in mitophagy-inducing conditions by nitrogen starvation in cells lacking either the *SLM35* gene or the protease-encoding *YME1* gene and looked for the appearance of lower-molecular-weight bands, which would correspond to the proteolytically edited form of 3HA-Atg32. *YME1* deletion caused the disappearance of a lower-molecular-weight band of 3HA-Atg32 (Fig. 3A, arrow in overexposure). However, *SLM35* deletion did not result in the accumulation of 3HA-Atg32 in either its processed or full-length form (Fig. 3A, normal exposure; Fig. 3B,C).

To rule out the possibility that we failed to detect an accumulation of the shorter cleaved version of 3HA-Atg32 due to its efficient degradation via mitophagy in cells lacking *SLM35*, we monitored 3HA-Atg32 protein levels in cells lacking the genes *PEP4* and *PRB1*, encoding vacuolar proteases (Fig. 3D). In this background, we could not observe a distinctive band appearing or disappearing in any of the strains, in other words, no shorter Yme1-dependent form of 3HA-Atg32 was detectable in the presence or absence of Slm35 or Yme1. Moreover, the full-length 3HA-Atg32 also remained unchanged in the absence of *SLM35* (Fig. 3E). Overall, these findings indicate that the effect of Slm35 on mitophagy is not due to increased levels or proteolytic editing of Atg32 by Yme1.

### Slm35 regulates mitophagy and oxidative stress resistance through mitochondrial retrograde signaling

Since our results indicate that Slm35 regulates mitophagy independently of Atg32 levels or its processing by Yme1, we next explored alternative mechanisms through which Slm35 may influence mitophagy. Given the reported link between RTG signaling and mitophagy (Journo et al., 2009), we investigated whether Slm35 exerts its effects via the RTG pathway. We hypothesized that Slm35 negatively regulates mitophagy by suppressing RTG activation. To test this, we monitored Cit2-GFP, a reporter of RTG activity, and Idh1-GFP, a mitophagy marker, in isogenic strains undergoing mitophagy induced by prolonged respiration in the stationary phase.

**Fig. 4.**
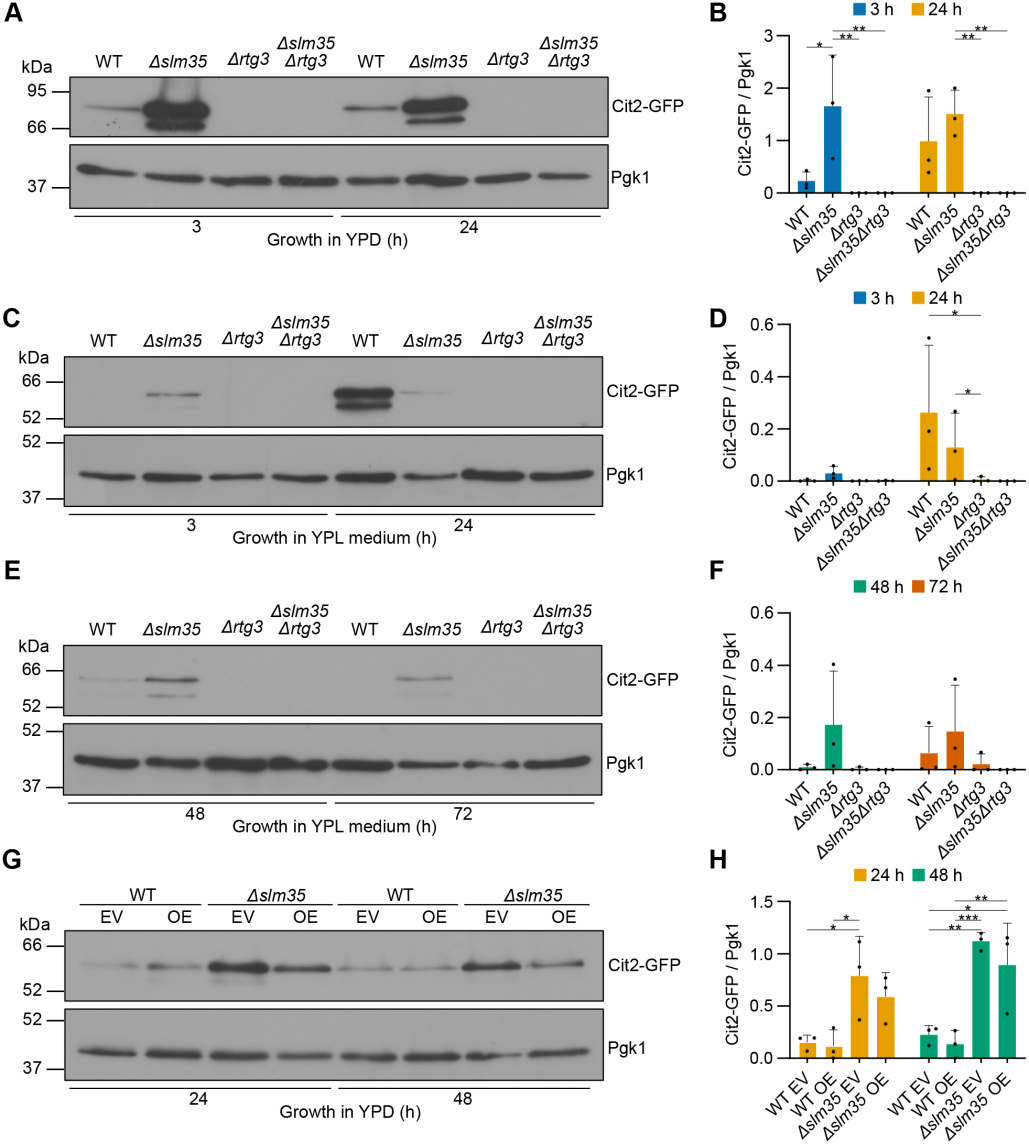
**Slm35 suppresses the RTG pathway activation.** (A) Cells expressing the *CIT2-GFP* RTG reporter were grown in YPD medium during exponential and stationary phases, and total protein extracts were collected at the indicated time points. Samples were analyzed by western blotting using antibodies against GFP and Pgk1. (B) Densitometric quantification of Cit2-GFP relative to Pgk1 signal from the blots in A. (C) Cells expressing the same reporter were grown in YPL medium, and protein extracts were collected after 3 and 24 h (early stages before mitophagy induction) and analyzed as in A. (D) Densitometric quantification of Cit2-GFP relative to Pgk1 signal from the blots in C. (E) Cells were grown in YPL medium for 48 and 72 h (late stages when mitophagy is established) and analyzed as in A. (F) Densitometric quantification of Cit2-GFP relative to Pgk1 signal from the blots in E. (G) Cells were grown in SD-Ura medium, and total protein extracts were collected at the indicated time points and analyzed as in A. (H) Densitometric quantification of Cit2-GFP relative to Pgk1 signal from the blots in G. Values represent mean±s.d. of *n*=3 independent biological replicates. Western blots in panels A, C and E were performed three times from independent replicates, and one representative blot is shown. Statistical significance was assessed using a two-way ANOVA followed by Tukey's multiple comparisons test. Significance levels: *P<0.05; **P<0.01; ***P<0.001. WT: wild-type strain. EV: empty vector. OE: *SLM35* overexpression (*pVT100U-SLM35-7HIS*).

Our results show that *SLM35* deletion enhances Cit2-GFP expression in a manner dependent on the Rtg3 transcription factor. This effect is observed during both exponential and stationary growth phases in glucose (Fig. 4A,B), as well as during prolonged growth on lactate – both at early time points before mitophagy induction (3 and 24 h, Fig. 4C,D) and at later stages when mitophagy is well established (48 and 72 h, Fig. 4E,F). In contrast, *SLM35*-*7HIS* overexpression did not cause a statistically significant change in Cit2-GFP levels (Fig. 4G,H).

**Fig. 5.**
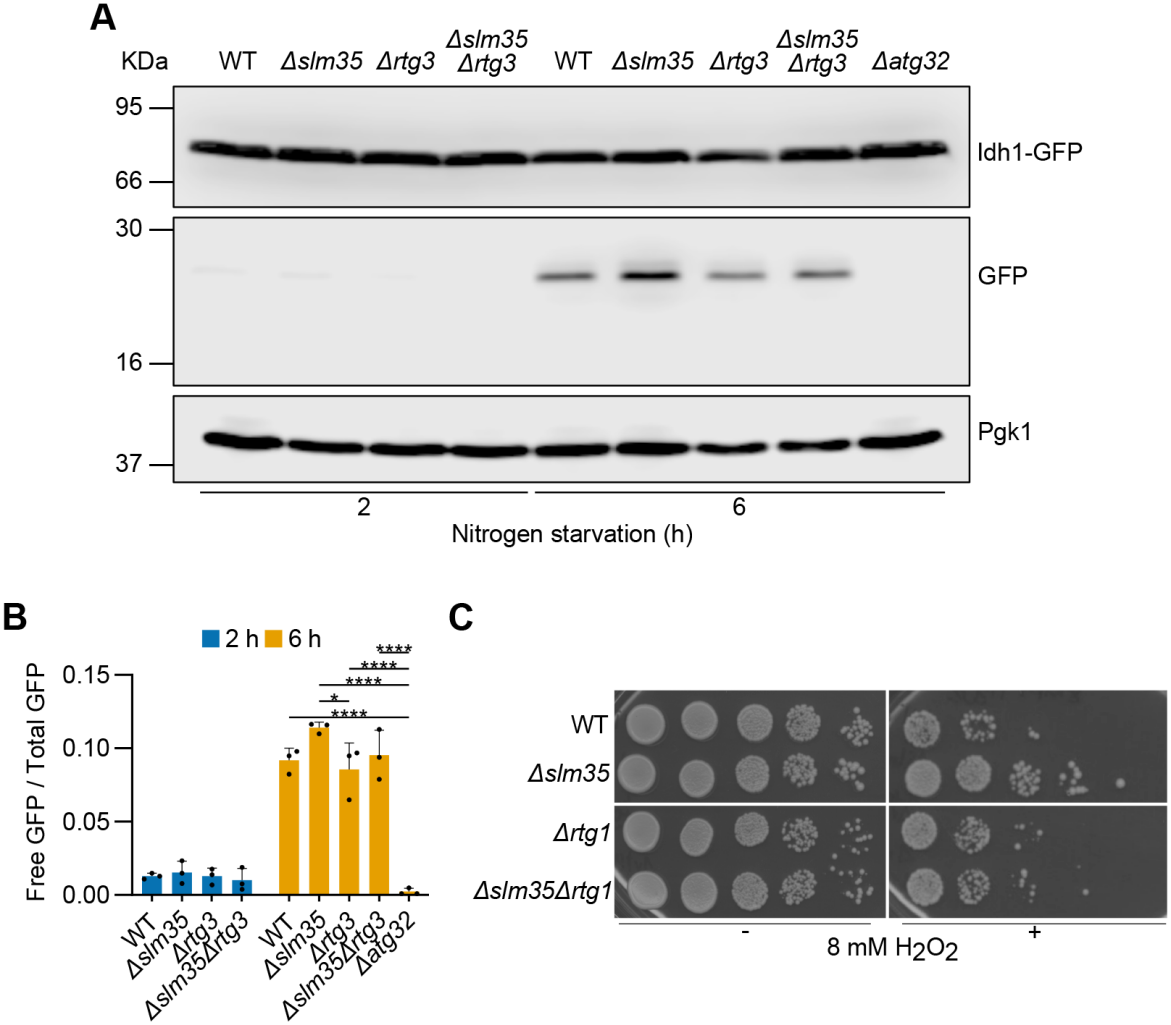
**Slm35's role in mitophagy depends on the RTG pathway.** (A) Cells expressing the *IDH1-GFP* mitophagy reporter were subjected to nitrogen starvation, and total protein extracts were collected at the indicated time points. Samples were analyzed by western blotting using antibodies against GFP and Pgk1. (B) Densitometric quantification of the free GFP relative to total GFP (Idh1-GFP+free GFP) signal from A. Values represent mean±s.d. of *n*=3 independent biological replicates. Statistical significance was assessed using a two-way ANOVA followed by Tukey's multiple comparisons test. Significance levels: *P<0.05; ****P<0.0001. Western blotting in A was performed three times from independent biological replicates, and one representative blot is shown. (C) Cultures of the indicated strains were treated with 8 mM H_2_O_2_ for 3 h (+) or left untreated (−), then serially diluted and spotted onto YPD plates. Plates were incubated at 30°C for 48 h.

Regarding mitophagy, *SLM35* deletion characteristically enhanced mitophagy, as indicated by an increased accumulation of free GFP compared to the wild-type strain. Conversely, *RTG3* deletion reduced the free GFP levels, confirming its role in mitophagy regulation. The double deletion mutant *Δslm35Δrtg3* shows free GFP levels similar to those observed in the wild-type strain and lower than those in the single *Δslm35* mutant (Fig. 5A). However, densitometric quantification revealed that these apparent differences were not statistically significant among the strains (Fig. 5B). Regarding mitophagy, the absence of *RTG3* in the presence or absence of *SLM35* (Fig. 5A), showed no statistically significant differences among the strains (Fig. 5B).

In addition to its role in mitophagy regulation, Slm35 has also been implicated in oxidative stress resistance by H_2_O_2_ (Aguilar-Lopez et al., 2016). Moreover, the RTG pathway itself has been linked to oxidative stress resistance (Guaragnella et al., 2019). Based on these reports, we hypothesized that the oxidative stress resistance characteristic of the *SLM35* mutant could be also related to the retrograde pathway. To test this, we performed growth serial dilution assays in which different deletion strains were exposed to H_2_O_2_ (Fig. 5C). Our results indicate that the oxidative stress resistance observed in the *SLM35* mutant is dependent on the RTG pathway, as the double deletion mutant *Δslm35Δrtg1* results in reduced growth, indicating an increased sensitivity to hydrogen peroxide.

### Slm35 does not alter mitochondrial membrane potential and growth in respiratory conditions

Since *SLM35* negatively regulates mitochondrial retrograde signaling and thus also mitophagy, we investigated whether *SLM35* influences the MMP, central for the activation of the RTG pathway in yeast (Miceli et al., 2012). To address this, we used Mitotracker™ Red FM or Rhodamine 123, mitochondria-specific dyes whose uptake depends on the MMP, and assessed via flow cytometry their fluorescence in bulk cell culture.

**Fig. 6.**
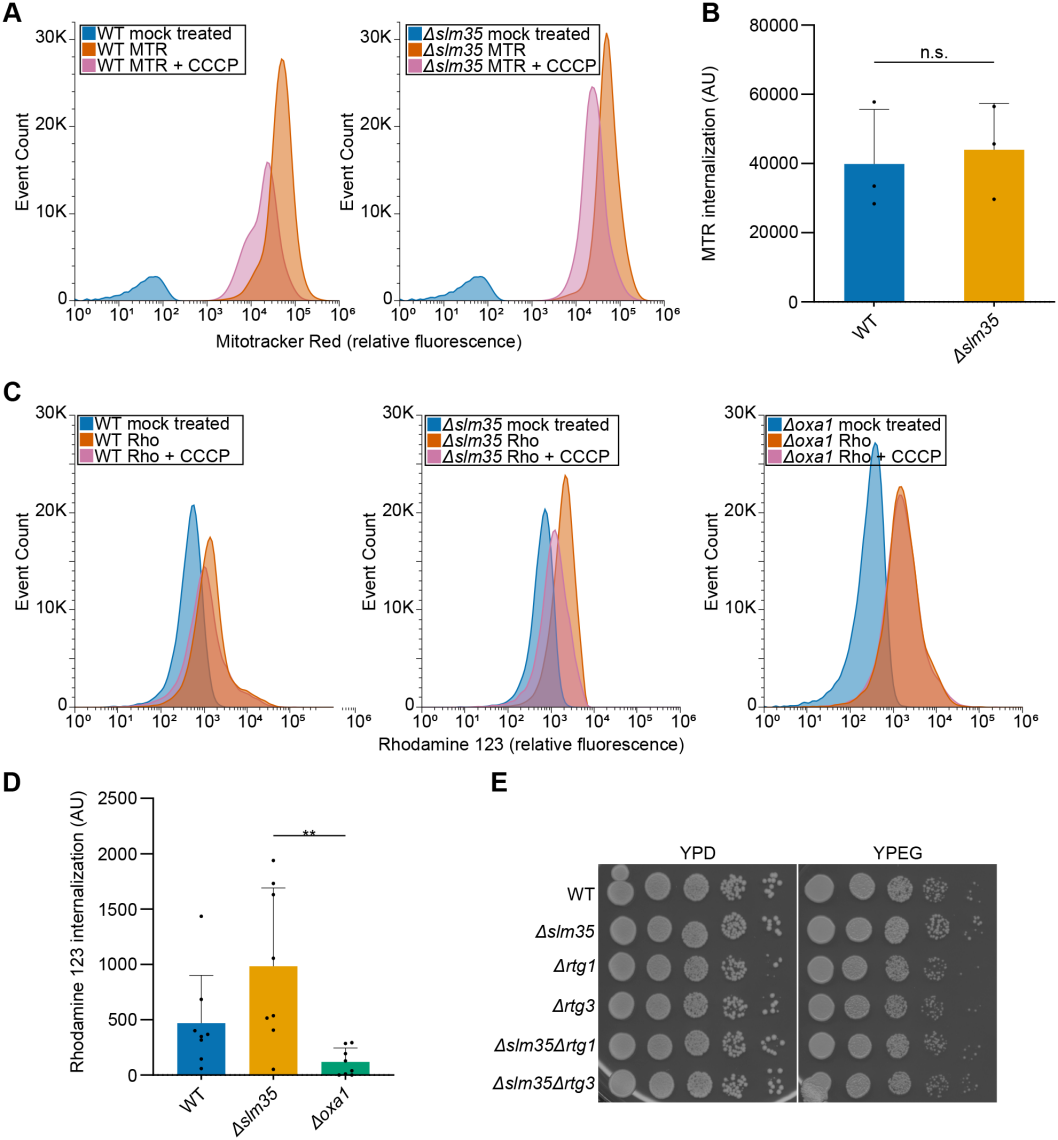
***SLM35* does not alter mitochondrial membrane potential.** (A) Flow cytometry histograms of cells grown to mid-log phase and stained with Mitotracker™ Red FM (MTR) alone or in combination with the mitochondrial membrane potential uncoupler CCCP, as indicated. (B) Relative MMP was calculated by subtracting the MFI of cells treated with MTR+CCCP (baseline) from that of cells treated with MTR alone. Data represent mean±s.d. (*n*=3 biological replicates per group). Statistical significance was determined using a two-tailed unpaired *t*-test. No statistically significant differences (n.s.; *P*≥0.05) were detected. (C) Flow cytometry histograms of cells grown to mid-log phase and stained with Rhodamine 123 (Rho) alone or in combination with CCCP, as indicated. (D) Relative MMP was calculated by subtracting the MFI of cells treated with Rho+CCCP (baseline) from that of cells treated with Rho alone. Data represent mean±s.d. (*n*=8 biological replicates per group). Statistical significance was determined using a one-way ANOVA followed by Tukey's multiple comparisons test. Significance levels: **P<0.01. (E) Yeast growth test by serial dilution assays of indicated strains taken from logarithmic phase cultures were spotted on rich fermentative (YPD) or respiratory (YPEG) plates and incubated at 30°C for 48 h (YPD) or 72 h (YPEG).

Using carbonyl cyanide m-chlorophenylhydrazone (CCCP) to collapse the MMP, thereby preventing the dye's uptake by mitochondria, we established a baseline fluorescence level for both wild-type and *Δslm35* cells (Fig. 6A,C). After correcting for this baseline, cells lacking *SLM35* did not exhibit any statistically significant differences in MMP compared to wild-type cells (Fig. 6B,D). Furthermore, cells lacking *SLM35* and those in which the RTG pathway is abolished grow normally in respiratory carbon sources (Fig. 6E). Since mitochondrial metabolism and MMP are critical for growth in these conditions, these findings suggest that the lack of Slm35 does not significantly alter mitochondrial bioenergetics.

## DISCUSSION

In this study, we show that Slm35 is a mitochondrial matrix soluble protein that downregulates both mitophagy and the RTG pathway. The combination of proteinase K protection assays and carbonate fractionation, suggests that Slm35-GFP behaves similarly to Ssc1, a well-characterized matrix soluble protein (Fig. 1A,B). This is further reinforced by structural prediction analyses, which reveal that Slm35 lacks the hydrophobic surface patches characteristic of integral membrane proteins (Fig. S1B). Additionally, the predicted structure of Slm35 retains the typical secondary conformation of tubby-like proteins (Fig. S1A) – a β-barrel enclosing a central hydrophobic α-helix (Boggon et al., 1999) – a structural motif also found in the phospholipid scramblase (PLSCR) family (Bateman et al., 2009). Unlike scramblases, Slm35 clusters with plant tubby-like proteins and lack an external transmembrane α-helix, a key feature required for intramembrane phospholipid translocation. Furthermore, no differences in lipid composition or scrambling activity have been observed in the absence of *SLM35* (Luévano-Martínez and Kowaltowski, 2017). Altogether, these findings indicate that Slm35 is a soluble mitochondrial protein, hence not involved in phospholipid metabolism.

Nevertheless, a previous study that performed a comprehensive proteomic analysis of integral, soluble, and membrane-associated protein fractions, categorized Slm35 as an integral membrane protein (Vögtle et al., 2017). Given our findings, this classification may have resulted from interactions with integral proteins in the IMM (Kohler et al., 2023; Lorenzi et al., 2016; Naumenko et al., 2017) or with mitoribosomes (Singh et al., 2020), which are known to maintain a tight interaction with the IMM (Zeng et al., 2018). Additional experiments, such as crosslinking or label-free identification methods, could clarify these interactions and confirm Slm35's matrix localization and interaction partners.

Our findings strongly suggest that *SLM35* negatively regulates mitophagy induced by different conditions (Fig. 2). Given that mitophagy serves as a quality control mechanism for mitochondria, its suppression by *SLM35* suggests a role in maintaining mitochondrial integrity under specific conditions. The partial rescue of mitophagy suppression by *SLM35* overexpression in the deletion strain underscores the complexity of its regulatory role and suggests that additional interacting factors may modulate its effects on mitophagy. While the Idh1-GFP processing assay used in this study provides a robust and widely accepted readout for mitophagy, complementary approaches could offer additional resolution. Future work employing mitophagy-specific assays, such as the mitochondrial phosphatase mtPho8Δ60 assay (Campbell and Thorsness, 1998; Kanki et al., 2009b) or the pH-sensitive fluorescent reporter Rosella (Rosado et al., 2008), which are already established in *S. cerevisiae*, as well as more recently developed systems like mt-Keima (Katayama et al., 2011), MitoTimer (Laker et al., 2014) and Mito-QC (McWilliams et al., 2016), would enable dynamic monitoring of mitochondrial turnover and provide further validation of the conclusions presented here.

How does *SLM35* regulate mitophagy? A key regulatory step in mitophagy initiation is the proteolytic processing of Atg32 by the mitochondrial protease Yme1 (Wang et al., 2013), making it a natural step for regulation. However, our data indicate that *SLM35* does not impact this processing step: deletion of *SLM35* did not cause an accumulation of either the full-length protein or the Yme1-processed form of 3HA-Atg32 (Fig. 3A-C), even when vacuolar degradation is inhibited to experimentally observe any possible protein accumulation (Fig. 3D,E). In line with this, other reports have suggested that Yme1-mediated proteolytic processing of Atg32 is not essential for mitophagy. For instance, N- or C-terminal tagging of Atg32 does not impair mitophagy, whereas truncation of the cytosolic domain abolishes the process (Levchenko et al., 2016). Moreover, C-terminally tagged Atg32 localizes correctly to mitochondria and rescues the mitophagy defect in *Δatg32* cells (Camougrand et al., 2020). Additionally, *Δyme1* cells exhibit near wild-type (Gaspard and McMaster, 2015; Welter et al., 2013) or even increased (Campbell and Thorsness, 1998) levels of mitophagy. Finally, C-terminally tagged Atg32 sustains its interaction with Atg11 (Onishi et al., 2023). These findings challenge the idea that Yme1 processing is essential for mitophagy initiation and instead highlight the importance of cytosolic signaling, particularly Atg32 phosphorylation. Although we were unable to assess the phosphorylation status of Atg32 in the absence of *SLM35*, the increased mitophagy observed in the deletion mutant raises the possibility that Slm35 could influence Atg32 phosphorylation through upstream signaling pathways. Further studies will be required to determine whether the kinases and phosphatase involved in Atg32 phosphorylation are indeed modulated by this functional network.

Another step for mitophagy regulation by *SLM35* may involve its connection to RTG signaling. Our results show that *SLM35* deletion increased the levels of the RTG reporter Cit2-GFP under various conditions (Fig. 4A-F), while *SLM35-7HIS* overexpression did not have a statistically significant effect on Cit2-GFP levels (Fig. 4G,H). However, our results do not rule out the possibility of an effect of Slm35 overexpression on RTG signaling under these specific conditions and further experiments will be needed to determine whether Slm35 directly influences RTG pathway activity or acts indirectly through changes in mitochondrial function. Interestingly, in prolonged respiration in the stationary phase, WT cells exhibited a distinct Cit2-GFP peak at 24 h, suggesting a controlled activation of the RTG pathway in response to nutrient depletion. In contrast, *SLM35*-deficient cells failed to show this transient peak, instead maintaining basal but persistent RTG activation (Fig. 4C,D). This deregulation implies that while WT cells can properly activate the RTG pathway to adapt to nutrient stress, *SLM35* deficiency disrupts this dynamic response, potentially altering mitophagy regulation. Our data suggest that the role of *SLM35* in mitophagy may involve the RTG pathway (Fig. 5A,B). This points to a potential role of the RTG pathway in regulating mitophagy during nitrogen starvation. Unlike previous findings (Journo et al., 2009), we observed mitophagy even when the RTG was abolished (Fig. 5A). The discrepancy with previous findings may stem from differences in experimental conditions, as Journo and colleagues examined the effect of *RTG3* on mitophagy induced by prolonged respiration in the stationary phase.

The RTG pathway is typically activated in response to mitochondrial dysfunction, specifically a decrease in membrane potential, rather than ATP depletion or reactive oxygen species accumulation, as shown in previous reports (Miceli et al., 2012). However, our findings rule out changes in MMP as a mechanism for Slm35's regulation of the RTG pathway and, therefore, mitophagy (Fig. 6A,B). Since mitochondrial metabolism and MMP are critical for growth in these conditions, these findings suggest that Slm35 does not directly influence mitochondrial bioenergetics. To further validate these findings, we assessed MMP using an independent dye, Rhodamine 123, in a larger dataset, which similarly showed no difference between the wild-type and *Δslm35* strains (Fig. 6C,D). Alternatively, one could argue that Slm35 might have a localized effect on a subpopulation of mitochondria that is not detectable in our bulk analyses. However, this scenario is unlikely, as the histograms do not reveal a distinct subpopulation of cells behaving differently upon *SLM35* deletion (Fig. 6A,C). Moreover, although the absence of *SLM35* has been reported to increase the loss of mtDNA (Hess et al., 2009) and this could also be related to a reduced mitochondrial membrane potential (Miceli et al., 2012), in our *Δslm35* mutants such decrease in membrane potential was not observed. This suggests that the overall mitochondrial function remains largely unaffected in the mutant population.

While mitophagy is typically associated with the degradation of dysfunctional mitochondria driven by a decrease of MMP or hypoxia-responsive mitophagy receptors (Bellot et al., 2009; Guo et al., 2001; Narendra et al., 2010; Okatsu et al., 2013), it has also been recognized for its physiological roles (Esteban–Martínez et al., 2017; Ordureau et al., 2021; Sandoval et al., 2008; Schweers et al., 2007). In this context, the role of Slm35 appears to be more metabolically driven rather than related to mitochondrial dysfunction. Yeast mitophagy does not directly respond to mitochondrial damage, as it is neither prevented by antioxidant activity (Deffieu et al., 2009) nor triggered by MMP dissipation (Mendl et al., 2011), ATP synthase inhibition (Kanki and Klionsky, 2008) or oxidative stress (Liao et al., 2020). Instead, yeast cells primarily activate mitophagy under nitrogen (Kanki and Klionsky, 2008) or glutathione restriction (Deffieu et al., 2009), suggesting a role in maintaining redox and nitrogen homeostasis. In particular, Deffieu et al. (2009) investigated the role of various amino acids in mitophagy regulation and found that only cysteine supplementation prevented its activation. One aspect that remains unclear is the potential role of glutamate, a metabolite known to negatively regulate the RTG pathway (Liu and Butow, 1999), which was not explicitly reported in their dataset. Since decreased glutamate levels can activate the RTG pathway, Slm35 may help maintain glutamate levels, thereby acting as a signal for mitophagy activation. Future studies measuring glutamate or directly supplementing it in Slm35-deficient cells could help clarify its potential role in linking the RTG pathway to mitophagy.

**Fig. 7.**
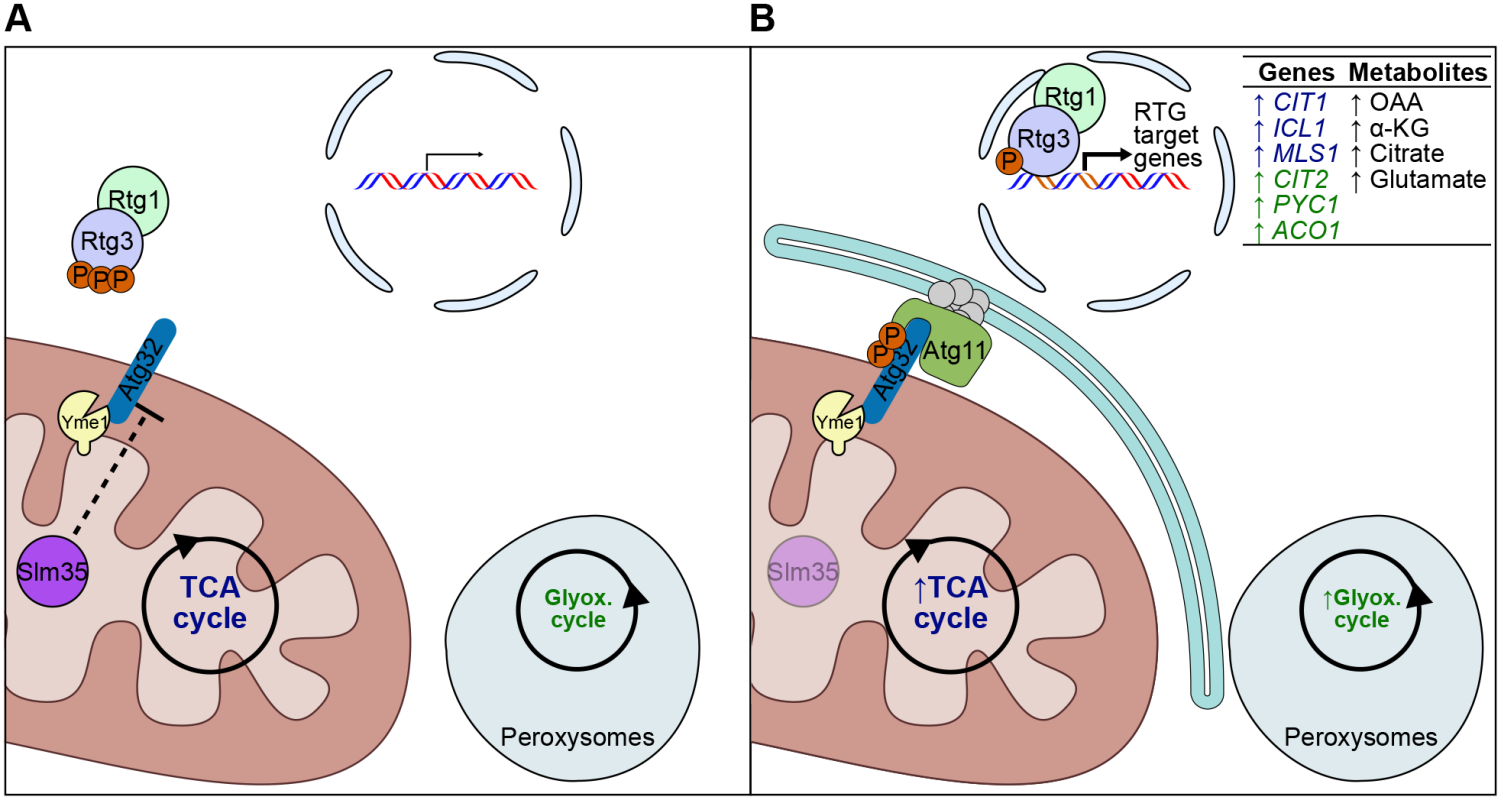
**Slm35 negatively regulates mitophagy and the RTG pathway.** (A) In wild-type cells, Slm35 localizes to the mitochondrial matrix, where it contributes to the regulation of Atg32, limiting mitophagy. The RTG pathway is transiently activated in response to metabolic stress but remains tightly controlled. Under these conditions, the TCA and glyoxylate cycles function efficiently. (B) In Slm35-deficient cells, mitophagy is enhanced, likely due to increased Atg32 phosphorylation and its interaction with Atg11, which recruits the autophagic machinery for phagophore formation. This process occurs independently of Yme1-mediated processing. The RTG pathway becomes constitutively active, as indicated by increased expression of *CIT2* and other RTG target genes, including those involved in the TCA and glyoxylate cycles (other than Cit2-GFP, not measured in this study). This may lead to increased activity of these metabolic pathways. Additionally, levels of metabolites produced by enzymes encoded by these genes may also be elevated (not measured in this study).

Together, our findings support a model in which Slm35 acts as a mitochondrial matrix protein that coordinately represses mitophagy and the RTG signaling pathway in *S. cerevisiae*. Under normal conditions, Slm35 may help to maintain Atg32 in a controlled, likely hypo-phosphorylated state, thereby limiting its interaction with Atg11 and preventing mitophagy initiation. Concurrently, Slm35 ensures tight regulation of the RTG pathway, enabling its transient activation in response to metabolic stress without sustained signaling. Under these conditions, the TCA and glyoxylate cycles would function efficiently (Fig. 7A). In the absence of Slm35, this regulatory balance is lost: Atg32 becomes hyperactive – potentially through increased phosphorylation – leading to enhanced recruitment of the autophagic machinery and elevated mitophagy, independently of Yme1-mediated processing. Simultaneously, RTG signaling becomes constitutively active. This is marked by the persistent upregulation of its target genes. Indeed, a comprehensive omics analysis (Stefely et al., 2016) found that protein levels of Cit2, a peroxisomal citrate synthase crucial for the glyoxylate cycle, as well as Cit1, Pyc1 and Aco1, enzymes of the TCA cycle, were all elevated in the absence of *SLM35* compared with the wild-type strain. Such increased expression of genes encoding enzymes for those cycles suggests an enhanced metabolic flux through these pathways, indicating a disruption in the cell's ability to modulate its metabolic response (Fig. 7B). Consequently, levels of metabolites produced by these mitochondrial TCA and peroxisomal glyoxylate cycle enzymes may also be elevated. Supporting this, the same study found that the levels of α-ketoglutarate, citrate and glutamate were also elevated. This model highlights a novel, bioenergetics-independent role for Slm35 in maintaining mitochondrial and metabolic homeostasis through dual regulation of mitophagy and retrograde signaling.

## MATERIALS AND METHODS

### Yeast strains, plasmids, and media

The *Saccharomyces cerevisiae* Meyen ex E.C.Hansen, 1883 strains used in this study (Table S1) were either obtained from the indicated reference or created by one-step PCR-mediated gene replacement, replacing the target gene open reading frames (ORFs) with antibiotic resistance or prototrophy modules with the plasmids in Table S2 and using the oligonucleotides from Table S3. Correct gene deletions were confirmed by PCR across the chromosomal insertion site.

To tag Cit2 with GFP at its carboxyl terminus in the mutants of interest, *CIT2-GFP-HIS3MX6* was amplified from the *CIT2-GFP* strain of the yeast GFP fusion collection (Huh et al., 2003) using the oligonucleotides listed in Table S3 and integrated into the nuclear genome of the BY4741 background strains through homologous recombination.

Unless stated otherwise, cells were grown in YPD medium [1% yeast extract (Gibco™), 2% peptone (Gibco™), 2% glucose (Sigma-Aldrich)] at 30°C. For selective growth or plasmid retention, cells were cultured in minimal synthetic medium (SMD) medium [0.17% yeast nitrogen base without ammonium sulfate or amino acids (Difco), (NH_4_)_2_SO_4_ 0.5% (Merck), 2% glucose (Sigma-Aldrich)] supplemented with leucine (0.01%), adenine (0.002%), histidine (0.002%), tryptophan (0.002%), methionine (0.002%), and lysine (0.003%) (all from Sigma-Aldrich). When used, G418 (Sigma-Aldrich), hygromycin (Sigma-Aldrich), and nourseothricin (Jena Bioscience) were added at final concentrations of 200, 250, and 100 µg/ml, respectively.

### Yeast growth test by serial dilutions

To assess the growth phenotype of the yeast strains in both fermentative and respiratory conditions, cells were first inoculated into YPD medium and incubated for 17-20 h at 30°C. Subsequently, cultures were diluted to the exponential phase [0.3 optical density at 600 nm (OD_600_)/ml] in fresh YPD medium and allowed to grow until reaching an optical density of approximately 1.0. At this point, a volume of culture corresponding to 1.0 OD_600_ was collected and serially diluted (1:10) in sterile water. For each dilution, 3.5 µl of each strain was spotted onto solid medium under the conditions specified in each experiment and incubated at 30 and 37°C.

To evaluate the resistance of the yeast strains to oxidative stress, cultures were grown and diluted to exponential phase (0.3 OD_600_/ml) YPD medium. Cells were exposed to oxidative stress by treating them with 8 mM hydrogen peroxide (Sigma-Aldrich) for 3 h. After treatment, serial dilutions of each culture, along with a non-treated control, were performed as previously described and spotted onto YPD plates. The plates were incubated at 30°C to assess growth recovery.

### Isolation of yeast mitochondria

Mitochondria were extracted from liquid cultures as previously described (Bauerschmitt et al., 2008). In brief, cells were collected by centrifugation (3000 ***g*** for 5 min) and washed with deionized water. MP1 buffer [100 mM tris-base (Sigma-Aldrich) and 10 mM dithiothreitol (Roche Diagnostics GmbH)] was added, incubated for 10 min at 30°C with shaking, and cells were precipitated by centrifugation (2000 ***g*** for 5 min). The cell pellet was washed with 1.2 M sorbitol and then resuspended in MP2 buffer [1.2 M sorbitol (Sigma-Aldrich) and 20 mM phosphate buffer (J.T.Baker), pH 7.4] along with 20 T zymolyase (2 mg/g wet weight; MP Biomedicals). This suspension was incubated at 30°C with shaking for 1 h to generate spheroplasts, which were collected by centrifugation (2000 ***g*** for 5 min at 4°C) and resuspended in MP3 buffer [0.6 M sorbitol (Sigma-Aldrich), 10 mM Tris-HCl (Sigma-Aldrich) pH 7.4, 1 mM EDTA (Sigma-Aldrich), 0.2% (w/v) bovine serum albumin (Roche Diagnostics GmbH), 1 mM phenylmethylsulfonyl fluoride (PMSF; Sigma-Aldrich)]. The spheroplasts in the suspension were mechanically lysed with 15 cycles in a pre-cooled glass homogenizer, and the cell debris was precipitated by centrifugation (2000 ***g*** for 5 min at 4°C). Mitochondria in the supernatant were collected by centrifugation (17,000 ***g*** for 12 min at 4°C) and resuspended in SH buffer [0.6 M sorbitol (Sigma-Aldrich), 20 mM HEPES (Sigma-Aldrich) pH 7.4].

### Protease protection and carbonate extraction

Protease protection assays were performed using crude mitochondria or mitoplasts incubated on ice in the presence or absence of proteinase K (PK), as previously described (Bauerschmitt et al., 2008). For intact mitochondria, 50 μg of crude mitochondrial protein extracts were diluted 1:10 in iso-osmolar SH buffer [0.6 M sorbitol (Sigma-Aldrich), 20 mM HEPES (Sigma-Aldrich) pH 7.4] to stabilize the organelles and incubated for 30 min on ice. Mitoplasts were generated by diluting 50 μg of crude mitochondria 1:10 in hypo-osmolar swelling buffer [10 mM HEPES (Sigma-Aldrich), pH 7.4] and incubating for 30 min on ice to promote outer membrane rupture. Where indicated, samples were treated with PK (50 μg/ml; Roche Diagnostics GmbH) for 30 min on ice. Complete membrane solubilization was achieved by adding Triton X-100 (Fisher Scientific) to a final concentration of 1%, when specified. Reactions were terminated by adding 400 μl of SH buffer containing 1 mM PMSF (Sigma-Aldrich) and 80 mM KCl (J.T.Baker). Samples were pelleted by centrifugation at 18,000 ***g*** for 10 min at 4°C, resuspended in Laemmli buffer (Laemmli, 1970) [60 mM Tris-HCl (Sigma-Aldrich) pH 6.8, 10% glycerol (J.T.Backer), 2% SDS (Sigma-Aldrich), 5% β-mercaptoethanol (Sigma-Aldrich), and 0.02% Bromophenol Blue (AMRESCO)], and subjected to western blot analysis on 14% polyacrylamide gels.

Carbonate extraction was carried out as previously described (Bauerschmitt et al., 2008). A total of 500 μg of isolated mitochondria was used per assay; 10% (50 μg) was set aside as the total fraction (T), and the remaining 90% (450 μg) were processed for carbonate extraction. The 450 μg of mitochondria were pelleted at 20,000 ***g*** for 10 min at 4°C and resuspended in 500 μl of freshly prepared ice-cold 100 mM Na_2_CO_3_ (J.T.Baker). Samples were incubated for 30 min at 4°C with vigorous shaking to release soluble proteins, followed by ultracentrifugation at 90,000 ***g*** for 20 min at 4°C to separate the soluble (supernatant; S) and membrane-associated (pellet; P) fractions. Proteins in the soluble fraction were precipitated by adding trichloroacetic acid (TCA; Sigma-Aldrich) to a final concentration of 12%, followed by washing with cold acetone (WÖHLER). Pellets from both fractions were resuspended in Laemmli buffer and subjected to western blot analysis on 14% polyacrylamide gels.

### Mitophagy assays

To induce mitophagy, two methods were employed. (1) By prolonged growth in a respiratory carbon source: cultures diluted in lactate or glycerol media, similar to as previously described (Kanki and Klionsky, 2008). Specifically, liquid cultures were grown in glucose medium (rich, YPD; or synthetic, SMD) and grown to stationary phase. The cultures were diluted to exponential growth phase (0.3 OD_600_/ml) in media containing 2% sodium lactate (Sigma-Aldrich) (YPL or SML) or 2% glycerol (J.T.Baker) (YPGly or SMGly) to induce mitophagy. Total protein extracts were collected at the indicated times in each experiment. (2) By nitrogen starvation: strains of interest were grown in glucose medium, diluted in lactate or glycerol medium for a few hours, and then the cells were transferred to nitrogen-restricted medium for 4 h to induce mitophagy (Guimaraes et al., 2015). Specifically, liquid cultures were grown in glucose medium (YPD or SMD) and grown to stationary phase. The cultures were diluted to exponential growth phase (0.2 OD_600_/ml) in 30 ml total of media with lactate or glycerol as carbon source (YPL, YPGly, SML or SMGly) until reaching an OD_600/_ml of 0.8-1.5. At that time, the cells from the cultures were collected by centrifugation (3609 ***g*** for 10 min), washed with sterile water, and resuspended in 30 ml of nitrogen-restricted medium SD-N [0.17% yeast nitrogen base without ammonium sulfate or amino acids (Difco), 2% glucose (Sigma-Aldrich)] to induce mitophagy. Protein extracts were collected at the indicated times of each experiment.

On both methods, mitophagy was evaluated by western blotting using total protein extracts to monitor the appearance of free GFP as a product of the proteolytic degradation of Idh1-GFP in the vacuole.

### Total protein extracts

A number of cells equivalent to 2 OD_600_ units was collected and washed via centrifugation at 8609 ***g*** using deionized water. The resulting cell pellet was resuspended in 250 µl of 50 mM Tris buffer (Sigma-Aldrich) (pH 8.0) and 50 µl of a lysis solution containing 0.3 N NaOH (J.T.Backer), 176 mM β-mercaptoethanol (Sigma-Aldrich), and 3.5 mM PMSF. The mixture was incubated on ice for 10 min to facilitate cell lysis. Proteins were precipitated by adding TCA (Sigma-Aldrich) to a final concentration of 12%, followed by incubation at −20°C. The precipitated proteins were washed with cold acetone (WÖHLER), after which the supernatant was completely removed. The protein pellet was subsequently dried and resuspended in loading Laemmli buffer.

### Western blotting

20 μg of total protein, or the amount of extract equivalent to 0.2 OD_600_ of culture cells per lane, was resolved by denaturing polyacrylamide gel electrophoresis (SDS-PAGE) at 120 V and 35 mA per gel in a vertical electrophoresis chamber. Proteins were transferred to an Amersham™Protran™ nitrocellulose membrane with a pore size of 0.2 μm (Cytiva) in a semi-dry chamber at 25 V and 400 mA for 1 h. Membranes were blocked with 5% skim milk for 1 h [overnight for anti-HA-HRP (horseradish peroxidase)] at 4°C and then incubated overnight at 4°C with the primary antibodies: mouse anti-GFP (Santa Cruz Biotechnology, sc-9996; 1:1000) for Idh1-GFP and free GFP; mouse anti-GFP (Roche, 11 814 460 001; 1:5000 for Cit2-GFP and 1:1000 for Slm35-GFP); mouse anti-Pgk1 (Thermo Fisher Scientific, 459250; 1:5000); rabbit anti-Ssc1(1:5000), anti-Oxa1 (N terminus; 1:5000) and anti-Tom20 (1:1000) antisera (kind gifts from Johannes M. Hermann's Laboratory). For 3HA-Atg32 detection, membranes were incubated for 2 h at room temperature with the peroxidase-conjugated anti-HA antibody (Roche, 12 013 819 001: 1:5000). Secondary antibodies used were anti-mouse (Jackson ImmunoResearch, 715-035-150; 1:10000) or anti-rabbit (Invitrogen, 31460; 1:5000). For His-tagged proteins, HisProbe™-HRP (Thermo Fisher Scientific, 15165; 1:5000) was used. HRP signal was detected by enhanced chemiluminescence using Immobilon Western Chemiluminescent HRP Substrate (Millipore, WBKLS0500). Detection was performed with either autoradiography or a C-DIGIT scanner (Li-COR Biosciences). Where indicated, band intensities were quantified using Fiji's (ImageJ 1.54g) gel analysis or Image Studio 5.1 (Li-Cor Biosciences).

### Mitochondrial membrane potential

Mitochondrial membrane potential was estimated by separate staining with MitoTracker™ Red FM (Invitrogen, M22425) or Rhodamine 123 (Invitrogen, R302), similar to what was previously described (Castillo-Casaña et al., 2025; Miceli et al., 2012; Volejníková et al., 2013). Briefly, the strains of interest were grown in glucose-rich medium (YPD) and then diluted to an OD_600_/ml=0.3 in SMD for 17 h. Cells equivalent to OD_600_=0.1 were collected, washed, and stained with MitoTracker™ Red FM (500 nM, 30 min at 30°C) or Rhodamine 123 (62.5 nM, 10 min at room temperature). Additionally, a separate aliquot was incubated with 10 μM CCCP (Sigma-Aldrich) for 10 min at 30°C before the addition of the dye to collapse the membrane potential and serve as a baseline fluorescence. Cells were centrifuged, washed three times with sterile water, and finally resuspended in 500 μl of sterile water for flow cytometry analysis.

Samples were analyzed using two different flow cytometers with dye-specific acquisition settings and no compensation applied. For MitoTracker™ Red FM experiments, the Attune NxT Acoustic Focusing Cytometer (Thermo Fisher Scientific) was used, acquiring data with forward scatter (FSC)=350 V, side scatter (SSC)=350 V, and YL2=350 V. For Rhodamine 123 experiments, an Attune Acoustic Flow Cytometer (Applied Biosystems) was used with FSC=3700 mV, SSC=4250 mV, and BL1=1500 mV. The excitation/emission parameters used were: FSC (488 nm), SSC (488/488 nm), YL2 (561/620 nm), BL1 (488/530 nm). The gating strategy involved defining the cell population using FSC-SSC and excluding doublets using an FSC-A vs FSC-H plot. The data reported corresponds to the quantification of the intensity of the MitoTracker™ Red FM or Rhodamine 123 signal on a logarithmic scale vs the count of the number of events (histogram). From this graph, the median fluorescence intensity (MFI) was obtained. To calculate the relative mitochondrial membrane potential (ΔΨ), the MFI of cells treated with either dye+CCCP (baseline) was subtracted from the MFI of cells treated with the dye alone. Data acquisition was performed using Attune™ NxT v3.2.1 (Thermo Fisher Scientific) and Attune Cytometric Software v1.2.5.3891 (Applied Biosystems) for MitoTracker™ Red FM and Rhodamine 123, respectively. Data were analyzed with the free online platform Floreada.io (accessed September 2025).

### Statistical methods

Densitometric quantification of band intensities was performed using Image Studio™ Software (Li-Cor Biosciences) or Fiji. Data are presented as mean±s.d. from the independent biological replicates, each derived from distinct cell populations, indicated in each experiment. Statistical significance was assessed in Prism 8 (GraphPad Software) using one way or two-way ANOVA followed by Tukey's multiple comparisons test, or two-tailed unpaired *t*-test. A *P*-value <0.05 was considered statistically significant.

### Use of AI tools

During manuscript preparation, generative AI tools (ChatGPT by OpenAI and Gemini by Google DeepMind) were used exclusively to assist with the reformulation and editing of text originally written by the authors. No AI tools were employed in the conception of experiments, data collection, analysis, figure preparation, or code generation. The authors remain fully responsible for the content of the manuscript.

## Supplementary Material

10.1242/biolopen.062106_sup1 Supplementary information
