# Supplementary material for: Retrograde signaling is required for Slm35-mediated negative regulation of mitophagy in yeast
Source: Biol Open. 2026 Jan 7;15(1):bio062106. doi: 10.1242/bio.062106 (PMC12817334; doi:10.1242/bio.062106)
Supplement: Supplementary information [file biolopen-15-062106-s1.pdf]

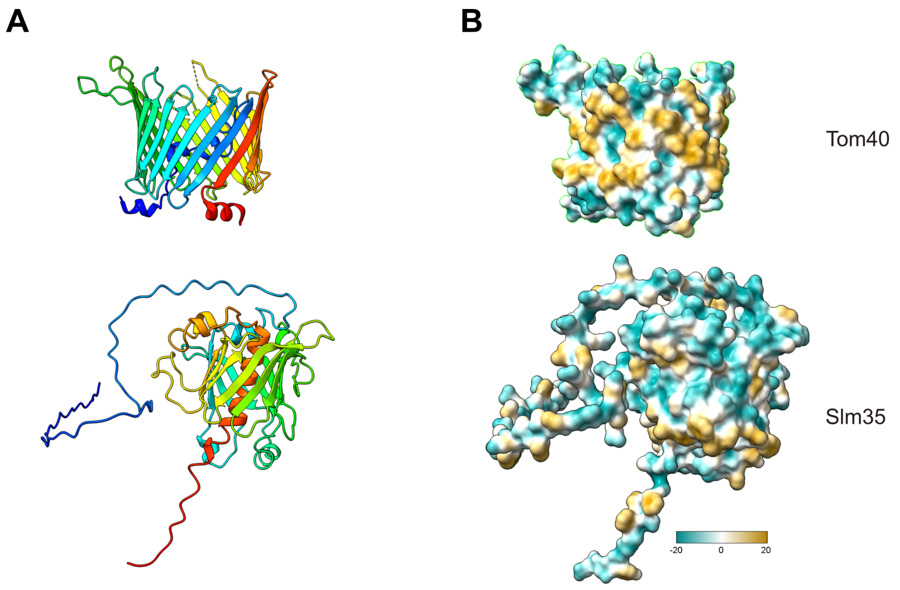

Fig. S1. Slm35 displays a β-barrel structure and lacks the hydrophobic patches found in Tom40. (A) Ribbon structure models of Tom40 (PDB 8W5J) and Slm35 (AlphaFold prediction, (Abramson et al., 2024)) reveal that Slm35 adopts a β-barrel structure, characteristic of OMM proteins rather than IMM proteins. (B) Surface hydrophobicity analysis highlights distinct hydrophobic patches (brown) in Tom40, which are absent in Slm35.

Table S1. Yeast strains used in this study

Strain Name	Genotype	Source
BY4741	*MATa his3Δ1 leu2Δ0 met15Δ0 ura3Δ0*	(Baker Brachmann et al., 1998)
BY4741 *Δslm35*	BY4741 *slm35::KanMX4*	(Aguilar-Lopez et al., 2016)
BY4741 *Δrtg1*	BY4741 *rtg1::KanMX4*	Euroscarf
BY4741 *Δslm35Δrtg1*	BY4741 *rtg1::KanMX4 slm35::hphMX4*	This work
BY4741 *Δrtg3*	BY4741 *rtg3::KanMX4*	Euroscarf

Biology Open • Supplementary information

Strain Name	Genotype	Source
BY4741 *Δslm35Δrtg3*	BY4741 *rtg3::KanMX4 slm35::hphMX4*	This work
BY4741 *Δoxa1*	BY4741 *oxa1::KanMX4*	Euroscarf
BY4741 *SLM35-GFP*	BY4741 *SLM35-GFP::HIS3MX6*	This Work
BY4741 *CIT2-GFP*	BY4741 *CIT2-GFP::HIS3MX6*	This work
BY4741 *CIT2-GFP Δslm35*	BY4741 *slm35::KanMX4 CIT2-GFP::HIS3MX6*	This work
BY4741 *CIT2-GFP Δrtg3*	BY4741 *rtg3::KanMX4 CIT2-GFP::HIS3MX6*	This work
BY4741 *CIT2-GFP Δslm35Δrtg3*	BY4741 *rtg3::KanMX4 slm35::hphMX4 CIT2-GFP::HIS3MX6*	This work
BY4741 *CIT2-GFP [pVT100U (empty)]*	BY4741 *CIT2-GFP::HIS3MX6 [pVT100U (empty)]*	This work
BY4741 *CIT2-GFP [pVT100U-SLM35-7HIS]*	BY4741 *CIT2-GFP::HIS3MX6 [pVT100U-SLM35-7HIS]*	This work
BY4741 *CIT2-GFP Δslm35 [pVT100U (empty)]*	BY4741 *slm35::KanMX4 CIT2-GFP::HIS3MX6 [pVT100U (empty)]*	This work
BY4741 *CIT2-GFP Δslm35 [pVT100U-SLM35-7HIS]*	BY4741 *slm35::KanMX4 CIT2-GFP::HIS3MX6 [pVT100U-SLM35-7HIS]*	This work
EY0986 (*IDH1-GFP*)	*MATa his3Δ1 leu2Δ0 met15Δ0 IDH1-GFP*::HIS3 *ura3Δ0* (S288C)	(Huh et al., 2003)
EY0986 (*IDH1-GFP*) *Δslm35*	EY0986 (*IDH1-GFP*) *slm35::KanMX4*	(Aguilar-Lopez et al., 2016)
EY0986 (*IDH1-GFP*) *Δyme1*	EY0986 (*IDH1-GFP*) *yme1::KanMX4*	This work
EY0986 (*IDH1-GFP*) *Δatg32*	EY0986 (*IDH1-GFP*) *atg32::KanMX4*	This work

Strain Name	Genotype	Source
EY0986 (*IDH1-GFP*) *Δrtg3*	EY0986 (*IDH1-GFP*) *rtg3::KanMX4*	This work
EY0986 (*IDH1-GFP*) *Δslm35Δrtg3*	EY0986 (*IDH1-GFP*) *slm35::KanMX4 rtg3::hphMX4*	This work
EY0986 (*IDH1-GFP*) *[pVT100U (empty)]*	EY0986 (*IDH1-GFP*) *[pVT100U (empty)]*	This work
EY0986 (*IDH1-GFP*) *[pVT100U-SLM35-7 HIS]*	EY0986 (*IDH1-GFP*) *[pVT100U-SLM35-7HIS]*	This work
EY0986 (*IDH1-GFP*) *Δslm35 [pVT100U (empty)]*	EY0986 (*IDH1-GFP*) *slm35::KanMX4 [pVT100U (empty)]*	This work
EY0986 (*IDH1-GFP*) *Δslm35 [pVT100U-SLM35-7 HIS]*	EY0986 (*IDH1-GFP*) *slm35::KanMX4 [pVT100U-SLM35-7HIS]*	This work
EY0986 (*IDH1-GFP*) *Δatg32 [pVT100U (empty)]*	EY0986 (*IDH1-GFP*) *atg32::KanMX4 [pVT100U (vacío)]*	This work
EY0986 (*IDH1-GFP*) *[pRS316]*	EY0986 (*IDH1-GFP*) *[pRS316]*	This work
EY0986 (*IDH1-GFP*) *[pRS316-3HA-ATG32]*	EY0986 (*IDH1-GFP*) *[pRS316-3HA-ATG32]*	This work
EY0986 (*IDH1-GFP*) *Δpep4Δprb1 [pRS316-3HA-ATG32]*	EY0986 (*IDH1-GFP*) *pep4::hphMX4 prb1::natMX4 [pRS316-3HA-ATG32]*	This work
EY0986 (*IDH1-GFP*) *Δpep4Δprb1Δslm35 [pRS316-3HA-ATG32]*	EY0986 (*IDH1-GFP*) *slm35::KanMX4 pep4::hphMX4 prb1::natMX4 [pRS316-3HA-ATG32]*	This work

Strain Name	Genotype	Source
EY0986 (*IDH1-GFP*) *Δpep4Δprb1Δyme1* *[pRS316-3HA-ATG32]*	EY0986 (*IDH1-GFP*) *pep4::hphMX4 prb1::natMX4 yme1::KanMX4 [pRS316-3HA-ATG32]*	This work

Table S2. Plasmids used in this study

Plasmid name	Description	Source
pFa6A	Vector for amplifying the G418-resistance module *KanMX4*	(Wach et al., 1994)
pAG25	Vector for amplifying the nourseothricin-resistance module *natMX4*	(Goldstein and McCusker, 1999)
pAG32	Vector for amplifying the hygromycin-resistance module *hphMX4*	(Goldstein and McCusker, 1999)
pVT100U (empy)	Empty yeast expression vector (2μ, URA3). GFP from pVT100-mtGFP (Westermann and Neupert, 2000) was removed using using BamHI	This work
pVT100U-SLM35-7HIS	Yeast expression vector (2μ URA3, ADH promoter and terminator)	This work
pRS316	Empty yeast expression vector (CEN/ARS, URA3)	(Sikorski and Hieter, 1989)
pRS316-3HA-ATG32	Yeast expression vector (CEN/ARS, URA3, ATG32 promoter and terminator)	(García-Chávez et al., 2024)

Biology Open • Supplementary information

Table S3. Oligonucleotides used in this study

Name	Sequence	Use
ATG32 delta F	ATGGTTTTGGAATACCAACAAAGGGAAGGTAAAGGATCCTccagctgaagcttcgtacgc	Amplify antibiotic resistance modules from pAG25, pAG32, pFa6a
ATG32 delta R	TTACAATAGAATATAACCCAGTGCCAAAATCCGATTAGATtcgatgaattcgagctcgtt	Amplify antibiotic resistance modules from pAG25, pAG32, pFa6a
PEP4 delta F	ATTTAATCCAAATAAAATTCAAACAAAAACCAAAACTAACccagctgaagcttcgtacgc	Amplify antibiotic resistance modules from pAG25, pAG32, pFa6a
PEP4 delta R	GGCAGAAAGGATAGGGCGGAGAAGTAAGAAAAGTTTAGCtcgatgaattcgagctcgtt	Amplify antibiotic resistance modules from pAG25, pAG32, pFa6a
PRB1 delta F	CAATAAAAAAACAAACTAAACCTAATTCTAACAAGCAAAGccagctgaagcttcgtacgc	Amplify antibiotic resistance modules from pAG25, pAG32, pFa6a
PRB1 delta R	AAGAAAAAAAAAAGCAGCTGAAATTTTTCTAAATGAAGAAtcgatgaattcgagctcgtt	Amplify antibiotic resistance modules from pAG25, pAG32, pFa6a
RTG1 delta F	AACACTAGATAGTGAACCAAAAGAAAGCACAACACCAAACccagctgaagcttcgtacgc	Amplify antibiotic resistance modules from pAG25, pAG32, pFa6a
RTG1 delta R	GGTTATCACAACATAGCAATAGTGAGAGTCAGAAGTACTTtcgatgaattcgagctcgtt	Amplify antibiotic resistance modules from pAG25, pAG32, pFa6a
RTG3 delta F	ATTTTTTGTCAGGCGAACCTACTTCTTAAATAAGTGAAGAccagctgaagcttcgtacgc	Amplify antibiotic resistance modules from pAG25, pAG32, pFa6a
RTG3 delta R	TTTTTCAAATTTAATTTTTTCCCGCTAATAAGACCATAAAtcgatgaattcgagctcgtt	Amplify antibiotic resistance modules from pAG25, pAG32, pFa6a
SLM35 delta F	CAAAACAAAAGAAGATTAAAGGTCAAACATAAAGGATAACACCGACcgtacgctgcaggtcgac	Amplify antibiotic resistance modules from pAG25, pAG32, pFa6a

Biology Open • Supplementary information

Name	Sequence	Use
SLM35 delta R	GTCAAATATGTATTTACA GAATTCTTTTAAATATATA ATTCACatcgatgaattcgagctcg	Amplify antibiotic resistance modules from pAG25, pAG32, pFa6a
YME1 delta F	ATAATACATTGTGGATAG AACGAAAACAGAGACGT GATAGcgtacgctgcaggtcgac	Amplify antibiotic resistance modules from pAG25, pAG32, pFa6a
YME1 delta R	GAGGTAGGTTCCTTCATA CGTTTAACTTCTTAGAAT AAAAatcgatgaattcgagctcg	Amplify antibiotic resistance modules from pAG25, pAG32, pFa6a
ATG32F-300	ACCGTCTGTCTAGAGCAT ATATC	Confirm deletion by PCR across the chromosomal insertion site
ATG32R+182	TAACCAAGTTCTGTCCCA TTG	Confirm deletion by PCR across the chromosomal insertion site
PEP4F-161	GAGAAGCCTACCACGTA AGG	Confirm deletion by PCR across the chromosomal insertion site
PEP4R+841	CGCCTAAACCGATACCTT CA	Confirm deletion by PCR across the chromosomal insertion site
PRB1F-257	GGCTTTCGGCTTTGGAA ATT	Confirm deletion by PCR across the chromosomal insertion site
PRB1R+544	GGTTGGGGATGATCTTGG AG	Confirm deletion by PCR across the chromosomal insertion site
RTG1F-352	TGCCATTGGTACTGTCTT CGTG	Confirm deletion by PCR across the chromosomal insertion site
RTG1R+494	AAGGAGCTCAACCTCTT TGCTG	Confirm deletion by PCR across the chromosomal insertion site

Biology Open • Supplementary information

Name	Sequence	Use
RTG3F-291	AGCCTTCTTATGAGCAACAGAA	Confirm deletion by PCR across the chromosomal insertion site
RTG3R+709	AACTTCCTGCACGAAATGACGA	Confirm deletion by PCR across the chromosomal insertion site
SLM35F-96	TTGTAAGCCTACCGGCAATAA	Confirm deletion by PCR across the chromosomal insertion site
SLM35R+984	CTACTCATCATAGCCACCG	Confirm deletion by PCR across the chromosomal insertion site
YME1F-418	CGAGGCCTCACAGATGCTAA	Confirm deletion by PCR across the chromosomal insertion site
YME1R+396	GAGACCACGTATTGCGGGTA	Confirm deletion by PCR across the chromosomal insertion site
HygR	GCAATCGCGCATATGAAATC	Confirm deletion by PCR across the chromosomal insertion site
KanR	GGCAGTTCCATAGGATGGCA	Confirm deletion by PCR across the chromosomal insertion site
NatR	GTTGTTTATGTTCGGATGTG	Confirm deletion by PCR across the chromosomal insertion site
CIT2-GFPF	CTCAAAACTTTTTGTTTTAA	Amplify CIT2-GFP-HIS3MX6 to integrate it into another strain
CIT2-GFPR	GAAAAATATGCAGAGGGGTG	Amplify CIT2-GFP-HIS3MX6 to integrate it into another strain
HindIII-MTS-SLM35 F	aagctTATGCATAGAACGGCAATATTTCTAA	Cloning SLM35-7HIS into pVT100U

Biology Open • Supplementary information

Name	Sequence	Use
SLM35-7HIS-XbaI R	ATTCTAGActaatggtgatggtgatggtggtgCTCATCATAGCCACCGCC	Cloning SLM35-7HIS into pVT100U

Supplementary references

Aguilar-Lopez, J. L., Laboy, R., Jaimes-Miranda, F., Garay, E., DeLuna, A. and Funes, S. (2016). Slm35 links mitochondrial stress response and longevity through TOR signaling pathway. *Aging* 8, 3255–3271.

Baker Brachmann, C., Davies, A., Cost, G. J., Caputo, E., Li, J., Hieter, P. and Boeke, J. D. (1998). Designer deletion strains derived from *Saccharomyces cerevisiae* S288C: A useful set of strains and plasmids for PCR-mediated gene disruption and other applications. *Yeast* 14, 115–132.

García-Chávez, D., Domínguez-Martín, E., Kawasaki, L., Ongay-Larios, L., Ruelas-Ramírez, H., Mendoza-Martinez, A. E., Pardo, J. P., Funes, S. and Coria, R. (2024). Prohibitins, Phb1 and Phb2, function as Atg8 receptors to support yeast mitophagy and also play a negative regulatory role in Atg32 processing. *Autophagy* 20, 2478–2489.

Goldstein, A. L. and McCusker, J. H. (1999). Three new dominant drug resistance cassettes for gene disruption in *Saccharomyces cerevisiae*. *Yeast* 15, 1541–1553.

Huh, W.-K., Falvo, J. V., Gerke, L. C., Carroll, A. S., Howson, R. W., Weissman, J. S. and O'Shea, E. K. (2003). Global analysis of protein localization in budding yeast. *Nature* 425, 686–691.

Sikorski, R. S. and Hieter, P. (1989). A system of shuttle vectors and yeast host strains designed for efficient manipulation of DNA in *Saccharomyces cerevisiae*. *Genetics* 122, 19–27.

Wach, A., Brachat, A., Pöhlmann, R. and Philippsen, P. (1994). New heterologous modules for classical or PCR-based gene disruptions in *Saccharomyces cerevisiae*. *Yeast* 10, 1793–1808.
